# An Y shaped tunnel to bypass a hemorrhoid band ligation scar during rectal endoscopic submucosal dissection

**DOI:** 10.1055/a-2604-8189

**Published:** 2025-06-18

**Authors:** Dana Bilous, Cătălina Vlăduț, Catalin Dutei, Daniela Elena Mihaila, Adrian Tulin, Elena Tianu, Mihai Ciocirlan

**Affiliations:** 1 “Carol Davila” University of Medicine, Bucharest, Romania; 2 “Prof. Dr. Agrippa Ionescu” Hospital, Bucharest, Romania; 3 Laurus Medical, Pitesti, Romania

Endoscopic submucosal dissection (ESD) has advanced significantly in the past decade, incorporating numerous technical enhancements. For colorectal ESD, the European Society of Gastrointestinal Endoscopy (ESGE) recommends the pocket-creation method when traction devices are not employed 1 . Extending and opening the pocket at the opposite side of the lesion creates a tunnel, enabling resection of very large colorectal lesions through single, double, or multiple tunnels 2 3 4 .

We present a 54-year-old woman referred for endoscopic resection of a large rectal laterally spreading tumor (LST). Notably, the anal side of the lesion exhibited a substantial fibrous scar from prior hemorrhoidal band ligation, extending near and beyond the dentate line ( Fig. 1 ). Two years earlier, she had undergone hemorrhoidal band ligation without a preceding colonoscopy.

**Fig. 1:**
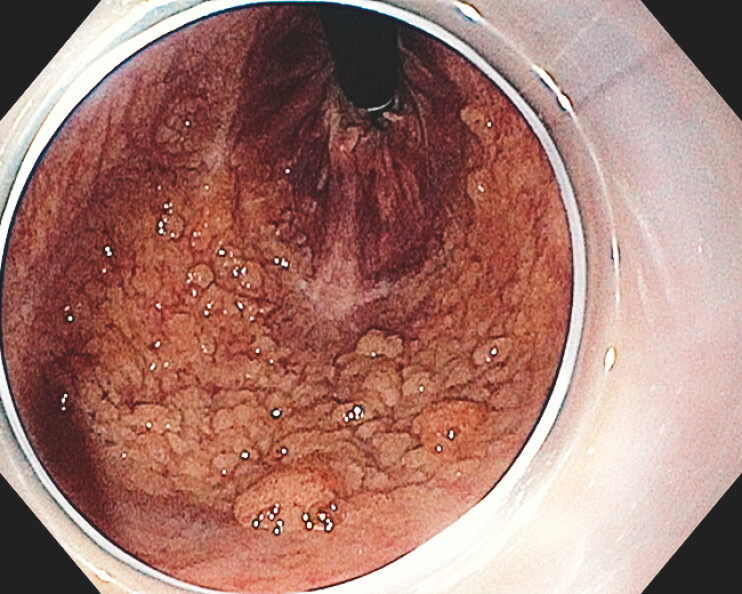
A large rectal laterally spreading tumor with a fibrous scar after hemorrhoidal band ligation near the dentate line.

To navigate the fibrotic area and achieve en-bloc resection, we employed a Y-shaped tunnel ESD technique ( Video 1 ). At the oral side, there was one opening (the base or the Y letter), while at the anal side, there were two openings (the oblique arms of the Y letter).

An Y-shaped tunnel seen from the oral side during rectal endoscopic submucosal dissection.Video 1

Minimal delayed bleeding occurred one week after the procedure, for which thermal coagulation was performed. Histopathological examination confirmed an R0 resection of a low-grade traditional adenoma.

While ESD of distal rectal LSTs overlying stapled mechanical hemorrhoidectomy sites has been documented, to our knowledge, this is the first reported case utilizing a Y-shaped tunnel to bypass a hemorrhoid band ligation scar during rectal ESD 5 .

Endoscopy_UCTN_Code_TTT_1AQ_2AD_3AZ

## Notes

Endoscopy E-Videos https://eref.thieme.de/e-videos
*E-Videos* is an open access online section of the journal *Endoscopy* , reporting on interesting cases and new techniques in gastroenterological endoscopy. All papers include a high-quality video and are published with a Creative Commons CC-BY license. Endoscopy E-Videos qualify for HINARI discounts and waivers and eligibility is automatically checked during the submission process. We grant 100% waivers to articles whose corresponding authors are based in Group A countries and 50% waivers to those who are based in Group B countries as classified by Research4Life (see: https://www.research4life.org/access/eligibility/ ). This section has its own submission website at https://mc.manuscriptcentral.com/e-videos .
